# The effect of steatosis and fibrosis on blunt force vulnerability of the liver

**DOI:** 10.1007/s00414-019-02245-4

**Published:** 2020-01-14

**Authors:** Gábor Simon, Viktor Soma Poór, Veronika Heckmann, Zsolt Kozma, Tamás F. Molnár

**Affiliations:** 1 Department of Forensic Medicine, Medical School, University of Pécs, 12 Szigeti street, Pécs, 7624 Hungary; 2 Department of Operational Medicine, Medical Humanities Unit, Medical School, University of Pécs, 12 Szigeti street, Pécs, 7624 Hungary; 3 Department Surgery, St Sebastian Thoracic Surgery Unit, Petz A University Teaching Hospital, 2-4 Vasvári Pál street, Győr, 9023 Hungary

**Keywords:** Forensics, Forensic pathology, Liver, Steatosis, Cirrhosis, Blunt force injuries

## Abstract

The aim of our study was to examine the possible effect of steatosis and fibrosis on the blunt force vulnerability of human liver tissue. 3.5 × 3.5 × 2-cm-sized liver tissue blocks were removed from 135 cadavers. All specimens underwent microscopical analysis. The tissue samples were put into a test stand, and a metal rod with a square-shaped head was pushed against the capsular surface. The force (Pmax) causing liver rupture was measured and registered with a Mecmesin AFG-500 force gauge. Six groups were formed according to the histological appearance of the liver tissue: intact (group 1), mild steatosis (group 2), moderate steatosis (group 3), severe steatosis (group 4), fibrosis (group 5), and cirrhosis (group 6). The average Pmax value was 34.1 N in intact liver samples (range from 18.1 to 60.8 N, SD ± 8.7), 45.1 N in mild steatosis (range from 24.2 to 79.8 N SD ± 12.6), 55.4 N in moderate steatosis (range from 28.9 to 92.5 N, SD ± 16.0), 57.6 N in severe steatosis (range from 39.8 to 71.5 N, SD ± 11.9), 63.7 N in fibrosis (range from 37.8 to 112.2 N, SD ± 19.5), and 87.1 N in the case of definite cirrhosis (range from 52.7 to 162.7 N, 30.3). The Pmax values were significantly higher in samples with visible structural change than in intact liver sample (*p* = 0.023, 0.001, 0.009, 0.0001, 0.0001 between group 1 and groups 2 to 6 respectively). Significant difference was found between mild steatosis (group 2) and cirrhosis (group 6) (*p* = 0.0001), but the difference between mild, moderate, and severe steatosis (groups 2, 3, and 4) was not significant. Our study demonstrated that contrary to what is expected as received wisdom dictates, the diseases of the parenchyma (steatosis and presence of fibrosis) positively correlate with the blunt force resistance of the liver tissue.

## Introduction

The liver is the most commonly injured abdominal organ in trauma [1, 2]. Traffic accidents account for the majority of liver injuries [3]. The incidence of traumatic liver injury in total population is 2.95–13.9/100.000 [4, 5]. Thirteen to 16% of polytrauma patients have liver injuries [6]. A direct frontal blunt impact usually causes the injury of the left liver lobe mostly along the falciform ligament (segments II, III, and IV), while impacts coming from lateral directions mostly affect the right lobe (segments V–VIII) [7].

Liver injuries can be caused by acceleration, deceleration, and compression/crush/ mechanisms [8]. The minimal impact velocities which can lead to liver injuries are predicted 5–8 m/s [9]. However, the mechanical vulnerability of tissues can show large individual differences, and these differences influence whether a blunt force results in an injury or not. The possible role of these individual differences has to be assessed in forensic situations many times.

The pathological changes of liver caused by diseases and/or dietary differences are very common. The fatty liver prevalence (alcoholic and non-alcoholic combined) is around 45% [10], and it increases with age. It is also common in children and young adults, reaching 17.3% for ages 15 to 19 years [11]. The estimated prevalence of hepatic fibrosis is around 3% [10].

Normal (healthy) liver contains 1–4% fibrous tissue, while cirrhotic contains 15–35% fibrous tissue. Normal human liver is estimated to contain approximately 5.5 mg/g of collagen, while cirrhotic liver contains approximately 30 mg/g [12]. Apart from the overall collagen content, type I/type III collagen ratio increases in cirrhotic liver above 20 mg of collagen/g [13]. Based on theoretical considerations, these structural changes should have a negative impact on the biomechanical properties and—more importantly in forensic aspects—on the vulnerability of the liver.

Textbook-based received wisdom suggests [14, 15] that certain diseases (e.g. steatosis) increase the vulnerability of liver, but no experimental data are available on the possible connection between pathological liver changes and blunt force vulnerability of human liver.

Biomechanical studies, compression, and strain test on liver samples have been performed by previous researchers [16]. These experiments used in vivo aspiration [17] or ex vivo methods [18–22] to define the mechanical properties of liver, and to create a mechanical resilience model for human liver [23]. However, no one of the previous studies examined the effect of different pathological conditions on the mechanical properties of liver samples. The degree of liver stiffness is determined by elasticity and viscosity. The liver matrix (collagen content) determines elasticity, while fatty infiltration, perfusion pressure, and inflammation determine viscosity [24]. A previous in vivo and ex vivo aspiration test on human liver suggested that increased connective tissue content increase the stiffness of liver, resulting in an increase of the stiffness index [25]. However, the results were inconclusive possibly due to the low number of samples. An animal study on rats indicated that chronic liver diseases increase liver stiffness [24].

The forensic pathologist is frequently challenged to evaluate the effect of preexisting liver diseases on blunt force vulnerability of the liver. The previous studies—mostly aiming to develop better diagnostic procedures—can offer only limited data.

Theoretically speaking, multiple factors can influence the blunt force vulnerability and resilience of the liver. These factors include liver size, liver weight, tissue density, tissue structure, capsule strength, and age. Liver size associated with steatosis may increase vulnerability [26], and larger organ weight causes larger forces during sudden deceleration, but a previous study suggested that steatosis does not increase the chance of blunt force liver injuries [26].

The aim of our study was to examine the possible effect of liver diseases on the vulnerability of liver tissue. A test system was developed and set up to emulate impact/compression type blunt force injuries of liver tissue on human liver samples using quasi-static blunt force.

## Materials and methods

### Samples

One hundred thirty-five liver samples were examined from human autopsy cases of the Department of Forensic Medicine, Medical School, University of Pécs. Prior to the autopsy, the bodies were stored at 4 °C from the onset of death, and no cadavers or samples were frozen. Previous freezing may interfere with the tensile properties and mechanical strength of tissues [27, 28]. Cases with unknown time of death or showing any macroscopic sign of putrefaction were excluded from the investigation. The cases who suffered previous traumatic liver injury, high energy impact (e.g. car accidents or falling from heights), poisoning, or had sepsis at the time of death were also excluded.

**Fig. 1:**
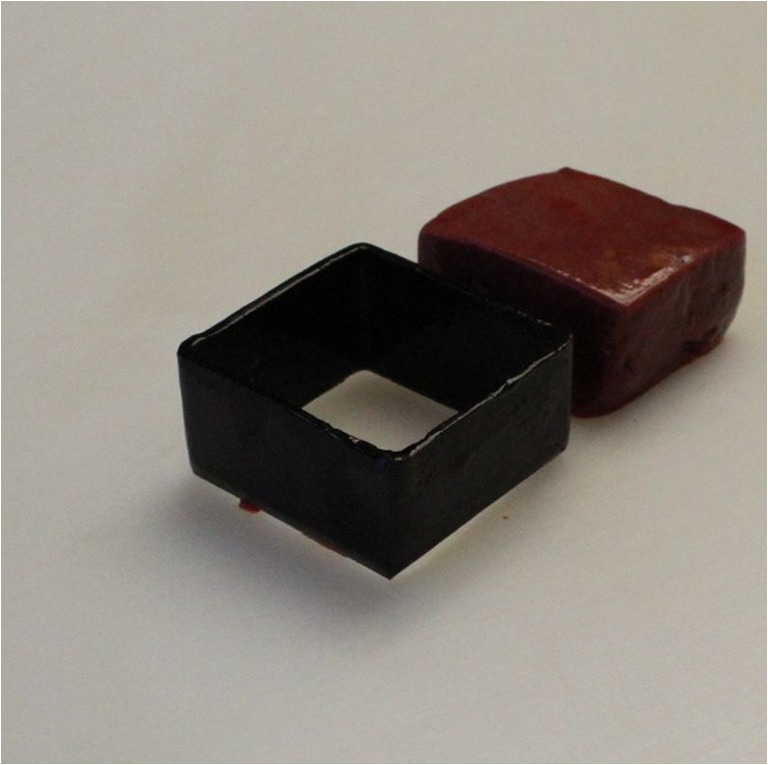
The rectangular metal frame used for removing the samples from the liver

The tissue blocks were removed from the anterior surface of the eighth liver segment with a 3.5 × 3.5 × 2-cm-sized rectangular metal frame (Fig. 1). The metal frame had a cutting edge allowing to take uniform-sized cubic-shaped samples. Considering its role in defending the liver parenchyma, the liver capsule was not removed from the tissue blocks.

### Mechanical tests

**Fig. 2:**
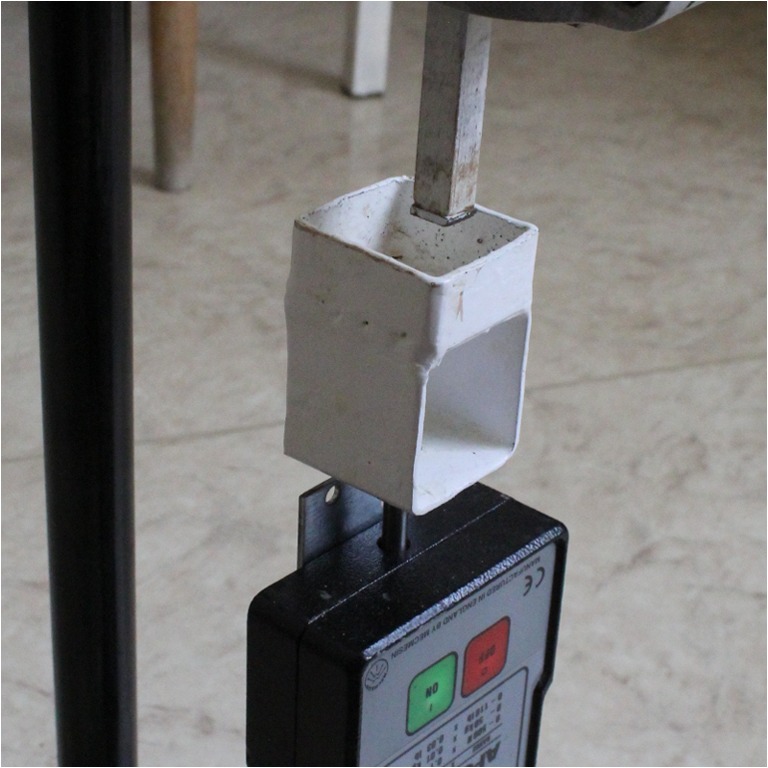
The force gauge and the test stand

The tissue blocks were positioned into a 3.5 × 3.5 × 2-cm-sized sample tray connected to the top of a Mecmesin AFG-500 force gauge (0–500 N measurement range, 0.1 N resolution). The force gauge and the sample tray were incorporated into a test stand (Fig. 2). The test stand was equipped with a downward facing rod with a square-shaped head with a 1-cm^2^-sized flat metal surface. A steadily increased pushing force has been applied on the capsular surface of the liver block. The breakthrough pressure resulting in the rupture of the capsule and lacerating the liver parenchyma was electronically registered as peak pressure (Pmax) by the force gauge.

### Tissue sample classification

**Fig. 3:**
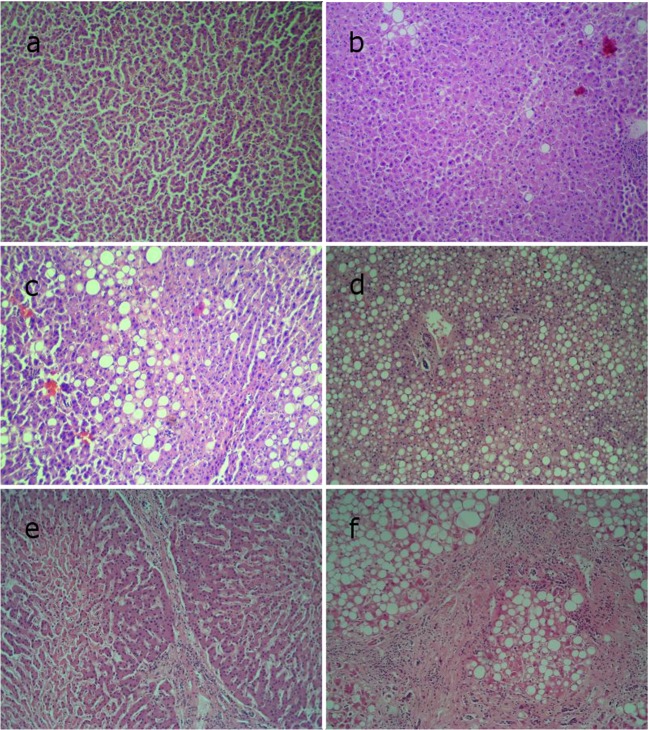
The six groups formed based on the histological appearance (HE). **a** Intact liver. **b** Mild steatosis. **c** Medium-grade steatosis. **d** Severe steatosis. **e** Fibrosis. **f** Cirrhosis

Histological samples were taken from each tissue block, and the histological appearance was evaluated under microscope using haematoxylin-eosin (HE) staining. Based on the microscopic appearance, six groups were formed using a modified liver steatosis and fibrosis classification [12, 25, 29] (Fig. 3a–f):

Group 1: intact liver samples without any visible microstructural change (no steatosis or fibrosis); (Int)

Group 2: mild steatosis (less than 1/3 of cells with steatosis); (Mil)

Group 3: medium-grade steatosis (1/3 to 2/3 of hepatocytes with steatosis); (Med)

Group 4: severe steatosis (more than 2/3 of hepatocytes with steatosis); (Sev)

Group 5: perisinusoidal, periportal, or bridging fibrosis, with or without steatosis; (Fib)

Group 6: liver cirrhosis (presence of nodules), with or without steatosis. (Cir)

Aetiology had no role in the selection process (i.e. alcohol induced vs other causes). Sixteen samples were excluded from the original study population because of microscopic signs of putrefaction, cellular (cancer or inflammatory cells), or foreign body infiltration. The average age of all cases included in the study was 58.72 years (SD ± 18.79; min-max, 4–100); 90 liver samples were obtained from males and 29 from females. The post-mortem interval (PMI) of liver samples ranged from 1 to 20 days (mean 7.32, SD ± 4.04).

## Statistical analysis

Statistical analysis was performed with SPSS 21 (IBM) statistical suit.

Multivariate analysis (Kruskal-Wallis test) was used for comparison of max force between groups. Where statistically significant difference (*p* < 0.05) was found, pairwise comparisons were performed to determine differences between relevant groups. In pairwise comparisons, significance levels were adjusted for multiple comparisons.

Relation between max force and age was tested with linear correlation. *R*^2^ was calculated. Level of significance was 0.05.

## Results

The groups (1–6) were proven comparable by age and sex. Forty-one liver samples showed no microscopic sign of structural change (group 1), 33 samples showed mild steatosis (group 2), 12 samples showed medium-grade steatosis (group 3), 6 samples showed severe steatosis (group 4), 11 samples showed fibrosis (group 5), and 16 definite cirrhosis (group 6). Most of the fibrotic and cirrhotic samples also showed some level of fatty infiltration. The registered Pmax values ranged from 18.1 to 162.7 N (average 50.41 N, SD ± 23.63).

**Fig. 4:**
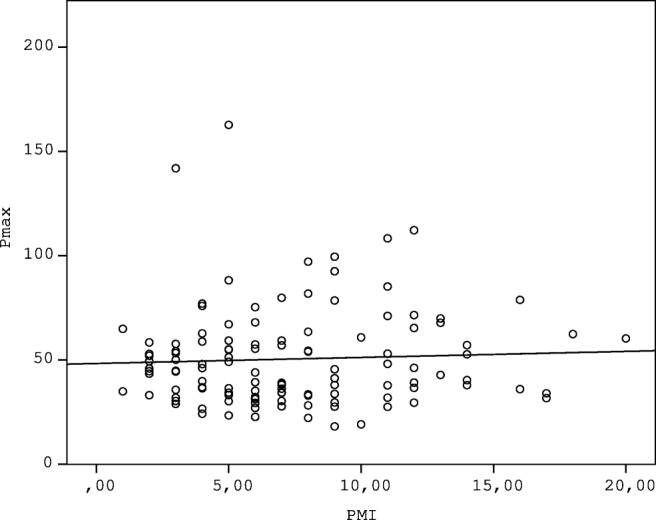
The figure indicates lack of correlation between Pmax (N) and PMI (days)

**Fig. 5:**
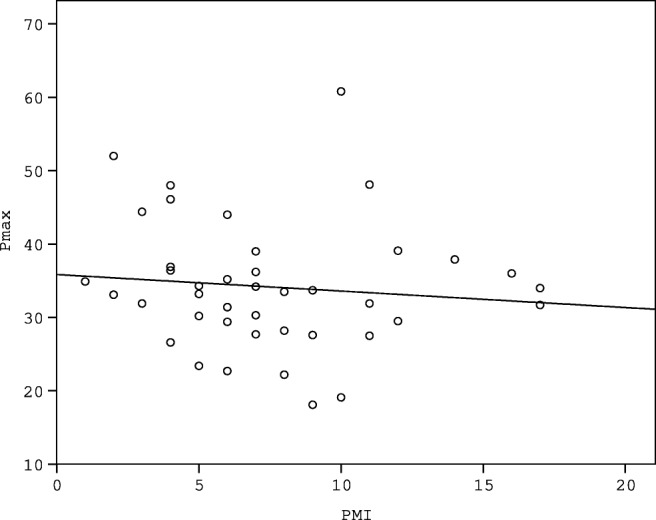
The figure indicates lack of correlation between Pmax (N) of intact samples (group 1) and PMI (days)

**Fig. 6:**
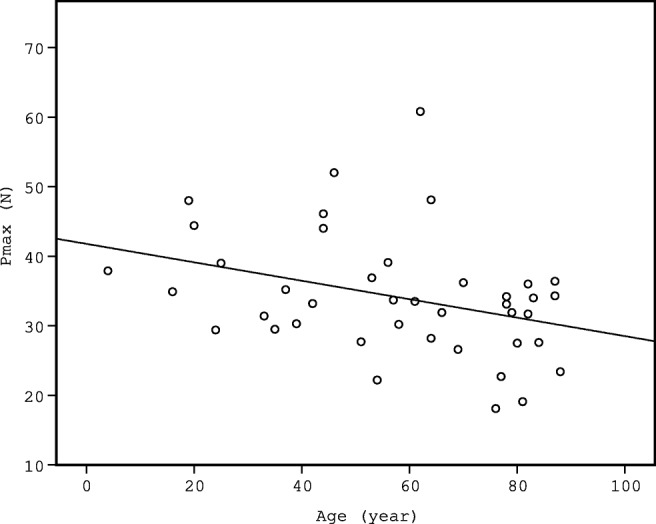
Correlation of Pmax (N) and age (years) in the intact group, showing the linear trend line. Linear correlation between the two parameters were significant (*p* = 0.025, *R*^2^ = 0.122)

The possible correlation between PMI and Pmax was analysed to assess the possible effect of PMI on blunt force vulnerability of liver tissue. No correlation was found between the PMI and the measured Pmax values (*p* = 0.630) (Fig. 4). No correlation was found between the PMI and Pmax in the intact liver group (*R*^2^ = 0.002 *p* = 0.592) (Fig. 5). The effect of age on liver vulnerability was also assessed, and age of the deceased in the intact group showed weak correlation with the Pmax values (*R*^2^ = 0.122, *p* = 0.025) (Fig. 6).

Multivariate regression analysis of the complete dataset did not reveal previously unidentified correlations with regard to the parameters evaluated. The histological feature-based classification strongly correlates with the Pmax (*p* < 0.001), while age and PMI have no significant effect on Pmax.

Age and PMI comparison of different histological groups showed no significant differences.

The average Pmax value was 34.1 N in intact liver samples, 45.1 N in mild steatosis, 55.4 N in moderate steatosis, 57.6 N in severe steatosis, 63.7 N in fibrosis, and 87.1 N in the case of definite cirrhosis (Table 1).

**Table 1:** Peak pressure at the time of laceration in different histological groups

Group	*N*	Age mean (range, SD±) (year)	PMI mean (range, SD±) (day)	Pmax mean (range) (N)	Pmax SD (N)
1. Int	41	57.4 (4–88, 22.98)	7.5 (1–17, 3.98)	34.1 (18.1–60.8)	8.7
2. Mil	33	54.5 (19–89, 17.15)	6.7 (2–20, 3.98)	44.6 (24,2-79,8)	12.6
3. Med	12	57.4 (28–70, 11.56)	6.3 (2–18, 4.84)	55.4 (28,9-92,5)	16.0
4. Sev	6	63.3 (55–70, 9.56)	6.5 (1–12, 4.50)	57.6 (39,8–71,5)	11.9
5. Fib	11	65.2 (33–100, 21.3)	7.7 (2–12, 3.43)	65.5 (37,8-112,2)	19.5
6. Cir	16	65.5 (44–91, 12.89)	8.8 (3–16, 4.03)	87.1 (52.76–162.7)	30.3

**Fig. 7:**
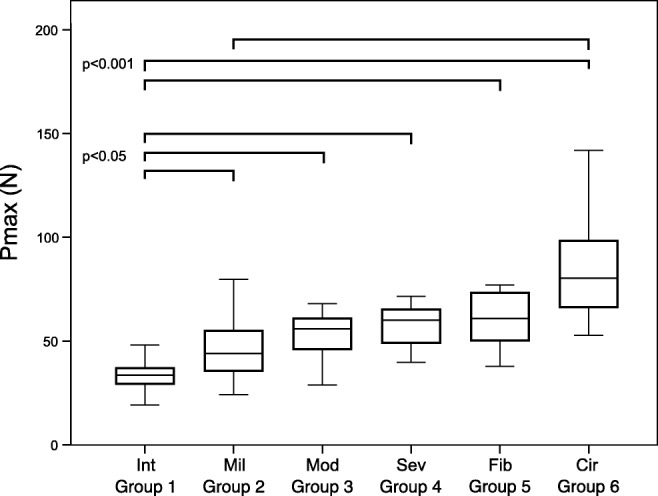
Pmax (N) values in the six histological groups. The Pmax values were significantly higher in samples with microscopic structural changes than in intact liver samples

The Pmax values were significantly higher in samples with microscopic structural changes than in intact liver samples (*p* = 0.023, 0.001, 0.009, 0.0001, 0.0001 between group 1 and groups 2 to 6 respectively). Significant difference was found between mild steatosis (group 2) and cirrhosis (group 6) (*p* = 0.0001). The difference between mild, moderate, and severe steatosis (group 2–4) was not significant (Fig. 7).

## Discussion

Our study showed that the steatosis, fibrosis, and cirrhosis decrease the blunt force vulnerability of the liver tissue. There is a clear-cut gradual increase in Pmax with progression of degree of pathologies. The mechanical properties of the fat tissue content and the increase of fibrotic tissue (collagen) explain the increased stiffness, as well as the increased tissue resistance in liver diseases with these structural changes. The experimental data also suggest that the vulnerability of liver tissue increases slightly by age, but the underlying histological condition is much more important determining the resistance to blunt force injury.

The data presented support statistical data from previous study [26]. The steatosis or fibrosis may increase the chance of liver injury due to the increased organ size and weight, but also increase the mechanical strength of the liver tissue.

The individual differences among the samples with similar histological appearance can be explained by multiple factors. The collagen content of tissues can differ slightly even when the histological appearance is similar, and the mechanical stiffness of the parenchyma can be masked by the stiffness of the capsule [25]. Diseases with microstructural changes affect the parenchyma to a larger extent than the capsule. Previous experiments proved that the capsule plays an important role in the mechanical strength of liver [30], and thickness and collagen content of the capsule can affect its mechanical stiffness [31]. The orientation of collagen fibres has also large effect on the biomechanical properties of the capsule [22], and therefore, it can be presumed that a similar effect is present in the parenchyma. In a living individual, also the actual blood perfusion can affect the mechanical properties [22, 23] due to its effect on viscosity [24]. Theoretically, the impact speed also might influence the vulnerability of the liver affected by different parenchymal diseases. The evaluations of the role of further factors like capsule structure, collagen content, impact velocity, and angle were beyond the scope of the present study and are matters of ongoing research awaiting for publication.

Our study using a compression-type blunt force in a quasi-static setting demonstrated that certain diseases of the parenchyma (steatosis, fibrosis) decrease the blunt force vulnerability of the liver tissue. The data contradict to the canonized teaching based on theoretical considerations as one may expect a more fragile, less mechanical stress-resistant liver of a cirrhotic patient. Previous statistical [26] and experimental data [24, 25] are supported by our data.

Our study gives useful data when the effect of structural diseases on liver vulnerability has to be assessed, but due to the limitations above explained, the effects cannot be quantified precisely. Further experiments assessing the role of overall collagen content of liver and the role of capsule thickness, capsule strength, and the use of dynamic forces can further detail, explain, and quantify the effect of pathological conditions on liver vulnerability.

In general terms, it can be stated with quite good certainty during a forensic evaluation of a blunt force liver injury that a given blunt liver rupture is not negatively related to the victims with previously existing hepatic steatosis or cirrhosis.
